# Persistence of EEG Alpha Entrainment Depends on Stimulus Phase at Offset

**DOI:** 10.3389/fnhum.2020.00139

**Published:** 2020-04-09

**Authors:** Mónica Otero, Pavel Prado-Gutiérrez, Alejandro Weinstein, María-José Escobar, Wael El-Deredy

**Affiliations:** ^1^Department of Electronic Engineering, Universidad Técnica Federico Santa María, Valparaíso, Chile; ^2^Advanced Center for Electrical and Electronic Engineering (AC3E), Universidad Técnica Federico Santa María, Valparaíso, Chile; ^3^Centro de Investigación y Desarrollo en Ingeniería en Salud, Universidad de Valparaíso, Valparaíso, Chile

**Keywords:** alpha entrainment, steady-state visually evoked potentials, alpha-band phase, alpha rhythm, persistence of the entrainment

## Abstract

Neural entrainment is the synchronization of neural activity to the frequency of repetitive external stimuli, which can be observed as an increase in the electroencephalogram (EEG) power spectrum at the driving frequency, -also known as the steady-state response. Although it has been systematically reported that the entrained EEG oscillation persists for approximately three cycles after stimulus offset, the neural mechanisms underpinning it remain unknown. Focusing on alpha oscillations, we adopt the dynamical excitation/inhibition framework, which suggests that phases of entrained EEG signals correspond to alternating excitatory/inhibitory states of the neural circuitry. We hypothesize that the duration of the persistence of entrainment is determined by the specific functional state of the entrained neural network at the time the stimulus ends. Steady-state visually evoked potentials (SSVEP) were elicited in 19 healthy volunteers at the participants’ individual alpha peaks. Visual stimulation consisted of a sinusoidally-varying light terminating at one of four phases: 0, π/2, π, and 3π/2. The persistence duration of the oscillatory activity was analyzed as a function of the terminating phase of the stimulus. Phases of the SSVEP at the stimulus termination were distributed within a constant range of values relative to the phase of the stimulus. Longer persistence durations were obtained when visual stimulation terminated towards the troughs of the alpha oscillations, while shorter persistence durations occurred when stimuli terminated near the peaks. Source localization analysis suggests that the persistence of entrainment reflects the functioning of fronto-occipital neuronal circuits, which might prime the sensory representation of incoming visual stimuli based on predictions about stimulus rhythmicity. Consequently, different states of the network at the end of the stimulation, corresponding to different states of intrinsic neuronal coupling, may determine the time windows over which coding of incoming sensory stimulation is modulated by the preceding oscillatory activity.

## Introduction

Neural entrainment refers to the synchronization of neural activity with the periodic properties of external stimuli. Entrainment can be elicited by driving forces such as magnetic or electrical fields generated by transcranial magnetic stimulation/transcranial alternating current stimulation, as well as rhythmic visual, auditory, and tactile sensory stimulation (Thut et al., 2011; Mathewson et al., 2012; De Graaf et al., 2013; Herrmann et al., 2016; Sameiro-Barbosa and Geiser, 2016).

Ideally, entrained oscillations arise from the realignment of the phase of ongoing oscillations to the driving stimulus. Consequently, entrainment can occur without power enhancement, if only the phase of the intrinsic oscillations is aligned to the external force. If, however, multiple oscillators are recorded in the electroencephalogram (EEG), their aligned phases add up and appear like an enhanced amplitude (Notbohm et al., 2016). Therefore, when periodic visual stimulation is presented, entrainment can lead to the generation of steady-state visually evoked potentials (SSVEP), i.e., scalp-recorded brain oscillations locked to the periodicity of the visual stimuli, which have relatively constant amplitude and phase over the stimulation interval. The SSVEP can be observed as an increase in the power spectrum at the frequency of the driving stimulus (Regan, 1966). Since SSVEP can also be explained by a series of evoked responses, the exact nature of the physiological mechanisms underlying this oscillatory evoked potential remains an active area of research. Nevertheless, evidence suggests that at least part of the SSVEP driven by visual stimulation in the alpha band of the EEG (8–14 Hz) can be attributed to the entrainment of alpha neural generators (Mathewson et al., 2012; Zauner et al., 2012; Spaak et al., 2014; Notbohm et al., 2016; Gulbinaite et al., 2017).

The amplitude of the stimulus-driven brain oscillation does not return to baseline immediately after the stimulus offset but remains relatively high for approximately three consecutive cycles, i.e., the entrainment persists after the stimulus offset (Sakamoto et al., 1993; Wacker et al., 2011; Halbleib et al., 2012; Mathewson et al., 2012; De Graaf et al., 2013; Spaak et al., 2014). This observation has been systematically reported. Nevertheless, the physiological mechanisms underlying the persistence itself has been scarcely described.

Most current hypotheses on the functional role of EEG oscillations agree that both induced and ongoing alpha waves represent a general inhibitory mechanism, which modulates perceptual and cognitive processes in the brain (Klimesch et al., 2007; Jensen and Mazaheri, 2010; Mathewson et al., 2011; Klimesch, 2012). This theory is based on studies of non-human primates, in which the increased action potential activity of somatosensory and motor cortical neurons during a vibrotactile discrimination task is preceded by a decrease in the power of ongoing alpha oscillations (Haegens et al., 2011). Furthermore, the reduced power of ongoing alpha oscillations has been associated with a higher probability of participants reporting sensory inputs regardless of the actual stimulus presence (Craddock et al., 2017; Iemi et al., 2017). Conversely, increased alpha power before the presentation of a target has been associated with low detection rates in visual discrimination tasks (Ergenoglu et al., 2004; Hanslmayr et al., 2007; van Dijk et al., 2008; Mathewson et al., 2009).

Alpha oscillations have been described as phasic changes that represent alternations between states of excitation/inhibition (Klimesch et al., 2007; Romei et al., 2007; Mathewson et al., 2011; Klimesch, 2012). This interpretation of alpha waves led to the prediction that identical visual stimuli may generate diverse perceptual representations, depending on the phase of the EEG oscillation at the moment of stimulus presentation (Lindsley, 1952; Busch et al., 2009; Mathewson et al., 2009; Milton and Pleydell-Pearce, 2016). The detection rate of brief visual stimuli and the identification of acoustic stimuli in a noisy environment strongly depend on the phase of ongoing alpha oscillations at the stimulus onset (Bishop, 1932; Dustman and Beck, 1965; Rice and Hagstrom, 1989; Busch et al., 2009; Mathewson et al., 2009). These results suggest that perception may operate in successive periodic cycles, alternating between phases of optimal excitability where stimuli are perceived, and phases associated with stronger inhibition at which the same stimuli are less likely to be detected (Klimesch et al., 2007; Busch et al., 2009; Schroeder and Lakatos, 2009; Milton and Pleydell-Pearce, 2016; Kizuk and Mathewson, 2017).

Similar to what has been observed in pre-stimulus intervals and during rhythmic visual stimulation, perceptual and cognitive processes derived from the presentation of a target has been linked to the phase and/or power of the alpha wave during persistence stage (Klimesch et al., 2003; De Graaf et al., 2013; Spaak et al., 2014; Kizuk and Mathewson, 2017; Ronconi and Melcher, 2017; Ronconi et al., 2018).

The persistence of the entrainment might represent the residual, post-stimulus activity of the SSVEP neural generators. Alternatively, it might reflect the functioning of cortical circuits actively coding the entrainer-offset, priming the sensory representation of incoming visual stimuli. Considering that different phases of alpha oscillations represent specific excitatory/inhibitory states in the brain, properties of persistence may depend on the phase of the brain oscillation at the stimulus offset (initial conditions of the persistence). Specifically, the duration of the persistence may vary as a function of the phase of the oscillation at the end of the stimulation. We addressed these hypotheses by eliciting SSVEP at the participant’s individual alpha frequency peak (IAF), which corresponds to the maximum power in the 8–14 Hz band of the ongoing EEG. Characterization of the persistence was carried out when SSVEP was elicited by continuous visual stimuli terminating at different phases. Specifically, we analyzed whether the duration of persistence depended on both the phase of the entrainer and the state of excitation/inhibition (i.e., the phase of the EEG signal) at the stimulus offset. The neural mechanisms underlying the persistence of entrainment were discussed based on source localization analysis of the different stages of the entrainment.

## Materials and Methods

### Participants

Nineteen healthy right-handed volunteers were recruited for the experiment (age = 26 ± 3 years; 11 male, eight females). Participants had normal or corrected to normal vision and reported no history of epilepsy, neurological, or psychiatric disorders. All the subjects gave written informed consent before participating. The experimental protocol was approved by the Research and Ethics Committee of the Universidad de Valparaíso (assessment statement code CEC170-18), in compliance with the national guidelines for research with human subjects and the Declaration of Helsinki.

### Experimental Design

**Figure 1:**
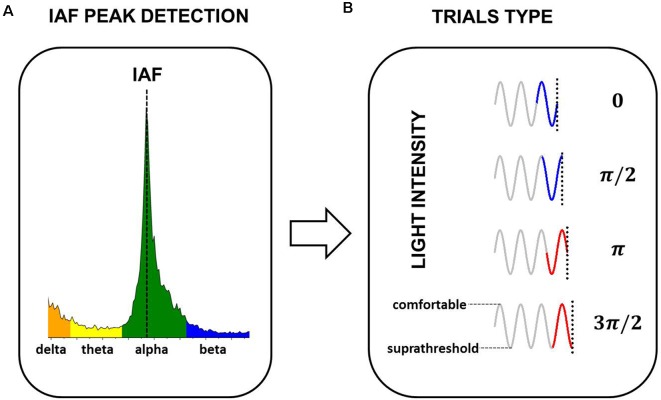
Experimental design. **(A)** Five minutes of EEG at rest were used to estimate Individual Alpha Frequency Peak (IAF) for each participant. **(B)** Sinusoidally-varying light with a frequency equal to the IAF of the participant was presented. Light varied between supra-threshold intensity and the maximum comfortable level of the participant. The phase of the sinusoid at the stimulus offset (0, π/2, π, and 3π/2) defined the four experimental conditions (four trial types). The light intensity at the stimulus onset always coincided with the mean intensity in the ascending ramp of the sinusoid (0 phase).

An overview of the experimental design is illustrated in Figure 1.

The peak frequency of brain oscillations in the 8–14 Hz frequency band was determined for each subject (IAF; Figure 1A). The IAF was used to modulate the intensity of the visual stimulation in the entrainment experiment. Light varied between a suprathreshold intensity and the maximum comfortable level of the participant in 200 current steps. The stimulus offset coincided with one of four possible phases of the sinusoid (Figure 1B, Trials Type). The technical details of the entrainment experiment are presented in “Entrainment Experiment” section. Stimuli were presented in three blocks of 60 trials each. Within each block, the four trials were counterbalanced and the order of presentation was randomized. The duration of the trials varied between 4.5 and 5 s, depending on the IAF of the participant and the experimental condition (phase of the stimulation at the offset). To avoid adaptation, the trials were followed by a resting interval of 2 s (Prado-Gutiérrez et al., 2019). Each block lasted between 6.5 and 7 min, with 1 min of rest in between. The entire experimental session lasted approximately 45 min.

### Estimation of the Individual Alpha Frequency Peak

The subjects sat in a comfortable chair, inside a dimly lit sound-attenuated and electromagnetically-shielded EEG chamber. Ongoing, eyes-open EEG was recorded for 5 min from 64 scalp locations using a radial electrode placement system (Biosemi Acquisition System). The signal was sampled at 8 kHz. Electrodes were also placed in periocular locations to record eye blinks and eye movements.

The EEG signal was pre-processed offline using standard procedures implemented in Brain Vision Analyzer 2.0^®^ (Brain Products GmbH, Munich, Germany). Recordings were re-referenced to the average of all channels, and band-pass filtered between 0.1 and 200 Hz using a zero-phase shift Butterworth filter of order 8. Data were downsampled to 512 Hz. Independent Component Analysis (ICA) was used for correcting EEG artifacts induced by blinking and eye movements (following Chaumon et al., 2015). Data were segmented into epochs of 5 s. The fast Fourier Transform (FFT) was calculated for each segment using a frequency resolution of 0.125 Hz, and the mean power spectrum was computed (Manolakis et al., 2000).

The IAF was calculated as the frequency corresponding to the maximum power spectrum value in the 8–14 Hz EEG frequency range (the peak frequency of brain oscillations in the alpha frequency band). Generally, this prominent peak in the EEG power spectrum is visible in the parieto-occipital electrodes when ongoing EEG is acquired (Berger, 1929). In this study, the maximum amplitude of the IAF, and the highest signal to noise ratio (SNR) of the recordings was systematically obtained in electrode Oz. Consequently, we chose the IAF computed in Oz for further analysis, which is following previous studies analyzing the ongoing alpha oscillations (Ronconi et al., 2018).

The IAF was used to set the frequency of the sinusoidally-varying light of the visual stimuli used in the main experiment. This was based on results demonstrating that entrainment can be optimal when the stimulation frequency of the entrainer lies within a narrow frequency range around the IAF (Pikovsky et al., 2003; Notbohm et al., 2016).

### Entrainment Experiment

For the entrainment experiment, the distance between the subject and a custom light-emitting diodes (LED) screen was adjusted to 70 cm. The position of the screen varied in the vertical direction and was adjusted to the eye-level of the participant.

The subjects were asked to look at the center of the LEDs screen, which consisted of four super bright 10 mm white LEDs of 16,000–20,000 mCd, and 30° of viewing angle^1^. LEDs were situated in the center of a 50 × 50 cm black screen, as vertices of a 5 × 5 cm square. The area of the square of LEDs subtended a visual angle of approximately 4°. The LED screen was designed using a Teensy 3.2 USB-based microcontroller development system. During stimulation, the light intensity of the four LEDs varied synchronously. The visual stimulation consisted of sinusoidally-varying light, i.e., a waxing and waning sinusoidal light source. The maximum intensity of the stimulation was settled at the maximum comfortable level defined by participants, and the minimum intensity was always suprathreshold. The frequency at which the intensity of the light was modulated corresponded to the IAF of the participant. The pulse width modulation (PWM) technique was used to control the power supplied to the LEDs. This technique allowed controlling the intensities of the LEDs at a given frequency, and generating the final sinusoidal envelope. The frequency of the PWM was set at 40 kHz to ensure reliable sinusoidally variations in the light intensity.

Four experimental conditions were defined, in which the stimulus-offset coincided with one of four possible phases of the sinusoid: 0; π/2; π and 3π/2 radians (Figure 1B). Importantly, the stimulus onset did not vary among experimental conditions, i.e., the stimulus always started at phase zero (mean intensity in the ascending ramp of the sinusoid). Therefore, the effect of the stimulus condition on the response cannot be interpreted as a consequence of the stimulus onset but must be explained as a consequence of the phase of the stimulation at the offset.

A procedure similar to that described by Dreyer and Herrmann (2015) was followed to validate the visual stimuli. The intensities of the LEDs were recorded using photodiodes (one photodiode for each LED on the display) at every stimulation frequency and every condition. The output of the photodiodes was recorded using a Saleae recording system (version Logic 8), using a sampling rate of 10 MHz. The signals were analyzed in both frequency and time domains to ensure that the expected frequency and waveforms were being driven. Specifically, we verified that the intensity of the light varied sinusoidally between the minimum and maximum values, that the frequency of the sinusoid matched the IAF, and that the stimulus indeed stopped at the target phases. In our simulation system, sinusoidally modulated lights which differed by 0.1 Hz were distinguishable.

The acquisition of the EEG during the stimulation and the corresponding pre-processing of the data was performed as described in the previous “Estimation of the Individual Alpha Frequency Peak” section. Since the amplitude of SSVEP is maximal over medial occipital electrode sites (Norcia et al., 2015; Vanegas et al., 2015), all further analyses were conducted using electrodes Oz and POz. In our recordings, these electrodes were the channels with the best SNR. They were also the channels at which the amplitude of the SSVEP was maximum. Instead of pulling the waveform of multiple electrodes, the signals acquired from Oz and POz were analyzed separately. This was based on the fact that averaging the waveform of multiple electrodes can reduce the overall effect, due to phase shifts between the channels (Notbohm et al., 2016).

**Figure 2:**
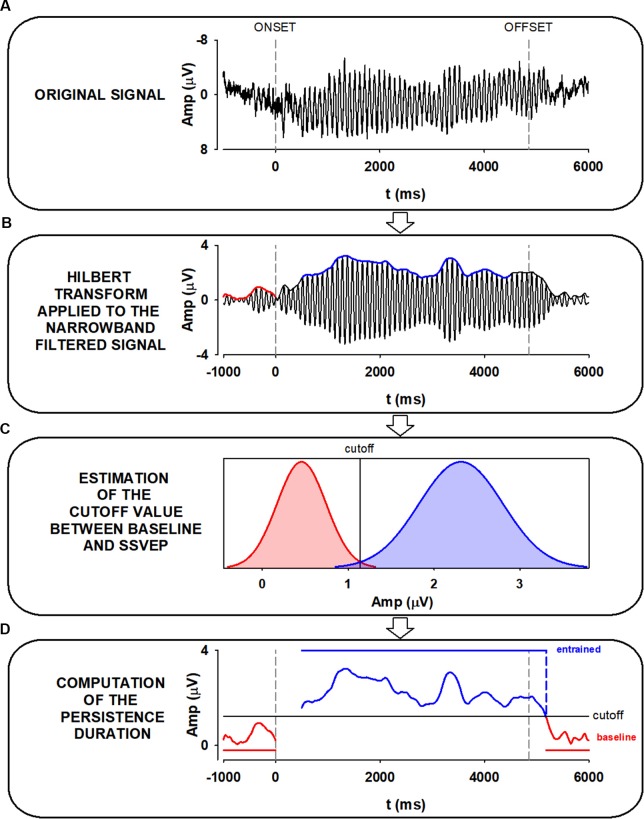
Pipeline for calculating the persistence duration (PD). **(A)** Steady-state visually evoked potentials (SSVEP) trials are averaged. Dashed vertical lines represent the stimulus onset and offset, respectively. **(B)** SSVEP trials are averaged and bandpass filtered ±1 Hz around the IAF. The envelope of the average SSVEP is extracted using the Hilbert transform, which results in an instantaneous estimate of the amplitude. **(C)** The distributions of envelope amplitudes in 1-s pre-stim (noise) and 4 s of entrainment (signal) are used to construct a probabilistic classifier to categorize the post-stimulus period as persistence of entertainment (signal distribution) or return to baseline (noise distribution). The vertical line marks the unbiased probabilistic boundary between pre-stimulus and entrainment (cutoff value). **(D)** Time points beyond stimulus offset are classified into entrained (blue) or not entrained (red) regions. Persistence duration is defined as the time from stimulus offset where the signal is continuously classified as belonging to the entrained distribution.

The EEG was segmented into epochs of 7 s. Epochs included 1 s of pre-stimulus activity, and 2 s of post-stimulus activity. Segmented signals were also DC-detrended, and baseline corrected. Forty-five trials belonging to the same experimental condition were time-domain averaged to increase the SNR of the measurements (Figure 2A). As a result, the SSVEP signals used for further analysis represented phase-locked brain oscillations across trials (Labecki et al., 2016; Prado-Gutiérrez et al., 2019).

A critical step in our study was obtaining an objective measure of the persistence duration (PD) of the oscillatory EEG activity after the stimulus offset. This can be achieved by computing the amplitude envelope of the oscillatory signal using the Hilbert transform. We use the Hilbert function provided in Matlab 2014a to obtain Discrete-time analytic signal. Moreover, the time resolution of the Hilbert transform was calculated. The Full-Width at Half-Maximum (FWHM) of the impulse response of the Hilbert transform corresponded to 7.8 ms.

Before obtaining the envelope of the signal *via* the Hilbert transform, the averaged signal was narrow-band filtered. This step is necessary as it has been previously demonstrated that the proper estimation of oscillatory parameters using the Hilbert transform can be performed only on narrow-band filtered signals (Huang et al., 1998; Chavez et al., 2006; Oweis and Abdulhay, 2011).

Therefore, averaged signals were narrow-band filtered between ±1 Hz around the IAF, using a zero-phase shift Butterworth filter of order 8, with a time constant of 0.018 s for a 10 Hz oscillation (Figure 2B). This procedure did not affect the evident oscillatory activity which succeeded the stimulus offset. Additional examples of the effect of the narrow-band filtered on SSVEP recordings are presented in Supplementary Figure S1.

For the analysis, the EEG signal was divided into three stages: baseline pre-stimulus (the 1,000-ms period just preceding the stimulus onset), SSVEP interval (4 s of the response, starting 500 ms after stimulus onset), and post-stimulus stage (from the stimulus offset until the time at which the oscillation returned to baseline; Figure 2C). The selection of the SSVEP interval was based on previous works indicating that a robust, stable entrainment is achieved after 400 ms of stimulation (Wacker et al., 2011; Halbleib et al., 2012; Salchow et al., 2016).

### Calculating Persistence Duration

The envelope of the signal was used to identify the boundary between the persistence of entrainment and baseline. We constructed individual classifiers based on the amplitude distribution of pre-stimulus (stimulus off, not entrained) and during stimulation (stimulus on, SSVEP; Figure 2C). This methodology followed the signal detection approach (Stanislaw and Todorov, 1999), where two conditions or distributions are discriminated: the “noise” distribution given by the baseline, and the “signal” distribution given by the entrainment. Gaussian functions were fit to the instantaneous amplitudes of pre-stimulus and SSVEP and the constructed normal distributions were used to calculate the probability of the post-stimulus amplitudes to belong to either baseline or entrained stages (Figure 2D).

The decision point (cutoff value for classification) was the intersection between the baseline and the entrained distributions (vertical line in Figure 2C). Therefore, the boundary between the persistence of entrainment and post-stimulus baseline (cutoff value) was defined using a neutral decision criterion, where neither stage was favored. The duration of the persistence of entrainment was defined as the time interval after the stimulus offset which was classified as belonging to the entrained stage, or conversely, that differed from baseline (Figure 2D). Persistence durations were compared using a two-way ANOVA, using “electrodes” (Oz vs. POz) and “phase at the stimulus offset” (0; π/2; π; 3π/2) as factors.

### Source Localization Analysis

Previous studies have suggested that occipital and frontal cortices interact during the generation of the SSVEP (Srinivasan et al., 2007; Halbleib et al., 2012; Xu et al., 2013; Li et al., 2015). To extend these results, the cortical areas involved in the persistence of the entrainment after the stimulus offset were estimated.

The neural sources generators of the scalp voltage distribution corresponding to the different stages of the SSVEP recording (baseline pre-stimulus period, SSVEP interval, and post-stimulus) were estimated using the Low-Resolution Electromagnetic Tomography method (LORETA, Pascual-Marqui et al., 1994; for a review, see Grech et al., 2008). LORETA calculates the current density at each of 6242 voxels in the cortical gray matter and the hippocampus of a reference brain (MNI 305, Brain Imaging Centre, Montreal Neurologic Institute) based on the linear, weighted sum of the scalp electric potentials.

Cortical activation maps were calculated for each of the scalp voltage distributions corresponding to the second of pre-stimulus activity just preceding the stimulus onset, the SSVEP interval (4 s of the response, starting 500 ms after stimulus onset), and the persistence of the entrainment (time interval between the stimulus offset and the time at which the oscillation returned to baseline, according to the output of the classifier described in Figure 2) of the narrow-band filtered data. Cortical activation maps corresponding to the same stage of the recording were averaged. Consequently, three maps were obtained for each participant, characterizing the baseline pre-stimulus period, the SSVEP, and the persistence of the entrainment.

The sample mean (*N* = 19) of the current density maps computed for the different stages of the SSVEP recording were pairwise compared using a *T*-test (*α* = 0.05). Multiple comparisons were corrected using a non-parametric, permutation test (20,000 randomizations).

## Results

### Neural Entrainment Elicited by Sinusoidally-Varying Visual Stimuli

**Figure 3:**
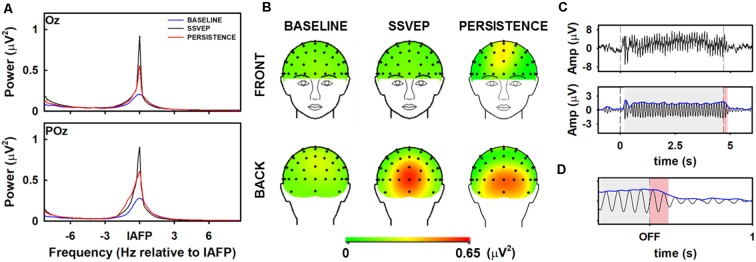
Spectral and spatial features of ongoing and stimulus-driven alpha oscillations. **(A)** Mean frequency spectrum of the EEG alpha band during pre-stimulus (blue), entrainment (black) and persistence (red) in electrodes Oz and POz. Spectral frequencies were centered on the individual alpha frequency peak (IAF). Power values were obtained by averaging the spectrum of all subjects (*N* = 19), in every experimental condition.** (B)** Mean topographic maps of alpha oscillations obtained by averaging the power spectrum of every subject (*N* = 19), during the pre-stimulus baseline stage (left panels), the SSVEP (center panels) and persistence (right panels). **(C)** Dynamics of the alpha oscillation. The traces represent the average of 45 trials, obtained in one volunteer. Top panel: unfiltered averaged data (EEG filtered between 0.5 and 200 Hz). Bottom panel: Narrowband filtered averaged data (±1 Hz around the IAF), and Hilbert transform (blue line). **(D)** Expanded view of the dynamic of the entrainment around the stimulus offset.

Across all 19 participants, the mean IAF (frequency at which the spectral peak of the ongoing EEG alpha band is obtained) was 10.1 ± 0.5 Hz (Figure 3A). The spectral power of the SSVEP was at least three times higher than that obtained in the pre-stimulus stage. As expected, the analysis of the topographic distribution of the SSVEP revealed that the increase in the power of alpha oscillations resulting from the visual stimulation was located in occipital scalp locations (Figure 3B). Maximum values of alpha power were obtained in electrodes Oz and POz.

The presentation of visual stimuli consisting of continuous, sinusoidally-varying light with frequency equals to the IAF elicited an onset-visual evoked potentials (onset VEP), which was followed by a gradual but moderate increase in the amplitude of the alpha oscillations (Figure 3C). The SSVEP was characterized by relatively stable amplitudes until the end of the stimulation. VEP was not evident at the stimulus offset. Oscillatory activity persisted for several periods of the oscillation after the stimulus offset (Figure 3D).

**Figure 4:**
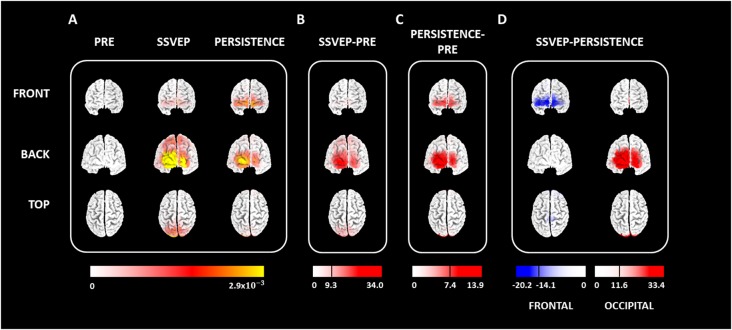
Cortical regions involved in the generation and persistence of the entrainment. **(A)** Mean cortical activation maps (*N* = 19) obtained for pre-stimulus, SSVEP, and the persistence stage. **(B)** The contrast between the SSVEP and pre-stimulus (SSVEP > pre-stimulus).** (C)** The contrast between persistence and pre-stimulus (persistence > pre-stimulus). **(D)** Contrast between SSVEP and persistence (SSVEP > persistence). Two regions of interest (ROI), corresponding to frontal and occipital cortices, were analyzed. Maps in** (B)** to **(D)** represent the corrected t-values resulting from the statistical test (*p* < 0.05, 20,000 permutations). In each case, the black line in the scale represents the threshold for statistical significance.

The source localization analysis (Figure 4A) revealed that the presentation of continuous sinusoidally-varying light elicited a statistically significant increase in the mean current density of cortical regions responsible for processing spectral, temporal and spatial visual information (contrast SSVEP > pre-stimulus: *t* = 9.36, *p* < 0.05; 20,000 permutations). Specifically, the SSVEP was associated with bilateral increased activation of Cuneus (Brodmann Area 17, BA 17) and the Middle Occipital Gyrus (BA 18), which correspond to the primary and secondary visual cortices, respectively (Figure 4B). Statistically significant activations were also obtained in other occipital, temporal and parietal locations, including the Inferior and Superior Occipital Gyri, as well as the Lingual and Fusiform Gyri (Supplementary Table S1).

Entrainment was found to outlast the stimulus offset (Figures 3C,D). In other words, the amplitude of the scalp-recorded alpha oscillation did not return to baseline immediately after the stimulus offset. Instead, the oscillations persisted for several 100 ms, in which the amplitude gradually decreased until the baseline. Across conditions, the duration of the persistence was 241.30 ± 119.96 ms. Since participants were stimulated at their own IAF, the persistent duration was also represented as the number of cycles of oscillatory activity. By combining all the experimental conditions among participants, the oscillation persisted for 2.43 ± 1.2 cycles.

The spectral power of alpha-oscillation computed in electrodes Oz and POz during persistence was higher than that obtained in the pre-stimulus stage but lower than that observed during the SSVEP (Figure 3A). As found in occipital locations, the power of alpha oscillations in frontal scalp locations was higher during persistence than in the pre-stimulus (Figure 3B). Nevertheless, oscillations computed in frontal electrodes were less powerful than those obtained in the occipital scalp locations.

Like the EEG oscillations recorded from the scalp, cortical activation associated with entrainment was not limited to the stimulation interval but persisted after the stimulus offset (Figure 4A). During persistence, significantly higher cortical activations than those computed during baseline were obtained (contrast persistence > pre-stimulus; *t* = 7.40, *p* < 0.05, 20,000 permutations). Increased activations were obtained in occipital areas, including the Cuneus, Lingual and Fusiform Gyri, as well as the Inferior, Middle and Superior Occipital Gyri (Figure 4C). Therefore, cortical regions generating the oscillatory activity after the stimulus offset were the same regions that were active during the visual stimulation. Noteworthy, when comparing the cortical activation maps between persistence and pre-stimulus, significantly higher activation was computed during the persistence in the Medial, Middle, and Superior Frontal Gyri (BA 10 and 11). Region with all statistically significant activations are presented in Supplementary Table S2.

Decreased activations in occipital regions, accompanied by increased current densities in the frontal cortex, became evident during the persistence of the entrainment as compared to those obtained during the SSVEP (Figure 4A). No statistically significant differences were found between these two stages of entrainment when mean activations were compared using a whole-brain analysis (*t* = 11.65, *p* = 0.063; paired *t*-test, 20,000 permutations). Consequently, *t*-test analyses were performed in selected regions of interest (ROI) to restrict the comparison to occipital and frontal locations (Figure 4D). Occipital ROI was composed of Cuneus, Fusiform, and Lingual Gyri, as well as Superior, Middle and Inferior Occipital Gyri. Frontal ROI included all voxels of the Inferior, Medial, Middle and Superior Frontal Gyri. When ROI-based analyses were performed, activation of the occipital cortex significantly decreased after the stimulus offset (contrast SSVEP > persistence: *t* = 11.61, *p* < 0.05, paired *t*-test, 20,000 permutations). By contrast, frontal regions displayed significantly higher activations during persistence than that computed during the SSVEP (contrast SSVEP > persistence: *t* = −14.18, *p* < 0.05, paired *t*-test, 20,000 permutations).

### Phase of Alpha-Oscillation at the Stimulus Offset Is Related to the Terminating Phase of the Visual Stimulus

**Figure 5:**
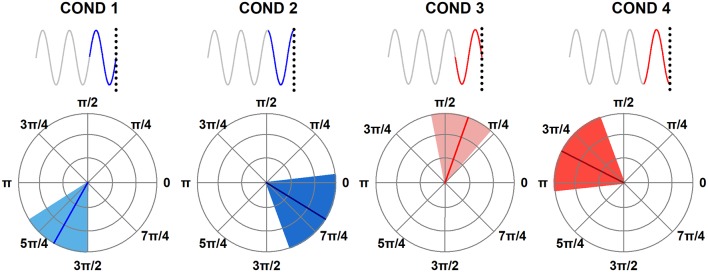
Phase angles of the EEG oscillation with respect to the ending phases of the stimuli for both Oz and POz electrodes taken together. Cond1, cond2, cond3, and cond4 correspond to the stimulus ending at phases 0, π/2, π and 3π/2 radians, respectively. Every stimulus condition ranges within a certain interval of angles. The lines on the graphs represent the mean direction for circular data. Blue or red shadows represent the upper and lower 95% confidence limits.

Using a similar procedure described by Mathewson et al. (2009), we calculated the phase angles of the EEG oscillation at the time the stimulus ended, for both Oz and POz electrodes. The mean direction and the circular standard deviation for circular data were calculated using the CircStat toolbox in Matlab (Berens, 2009). It was found that phases of the EEG signal at the stimulus termination were distributed within a constant range of values relative to the phase of the stimulus offset (Figure 5). This result was expected, since the phase lag between the stimulus and the SSVEP is constant (Regan, 1966; Nakanishi et al., 2014).

More importantly, two groups of EEG phases were distinguishable, describing the peak and troughs of the oscillation. EEG phases toward the trough of the alpha-oscillation were obtained when the terminating phase of the visual stimulation was zero, and π/2. Conversely, EEG phases toward the peak of the alpha-oscillation were observed when stimuli ended at π, and 3π/2. The same distribution of phases has been obtained in studies demonstrating that perception of transient stimuli is facilitated or impeded when stimuli are presented towards the troughs or peaks of the alpha-oscillation, respectively (Mathewson et al., 2009; Klimesch, 2012). Consequently, based on the distribution of phases of the alpha-oscillation at the time the stimulus ended, we grouped the termination phase of the visual stimuli in rising phases (zero and π/2, which corresponded with EEG phases towards the troughs of the alpha-oscillation), and falling phases (π and 3π/2, which corresponded with EEG phases towards the peaks of the alpha-oscillation).

### Persistence Duration Depends on the Stimulus Phase at Offset

**Figure 6:**
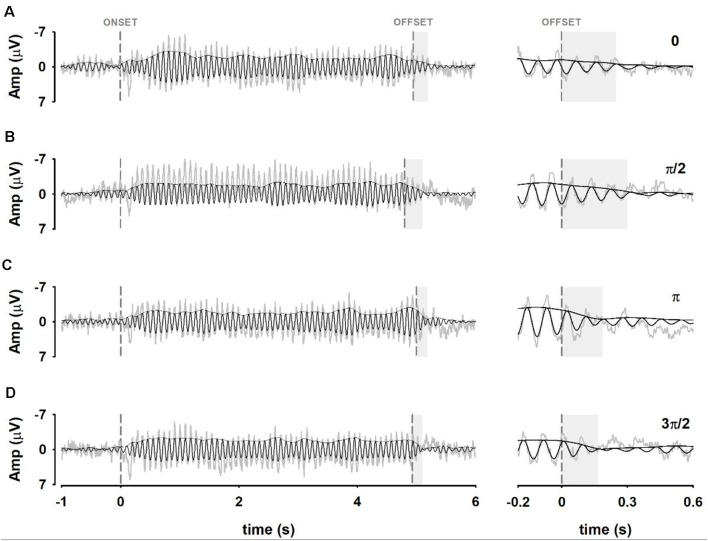
Waveforms of the neural entrainment obtained from electrode Oz in one representative subject. Panels **(A–D)** show waveforms of averaged EEG signals when the terminating phase of the stimulus was 0, π/2, π, 3π/2, respectively. (Left) Averaged EEG signals (light gray traces), narrow-band filtered signals (black traces) and envelopes calculated from the Hilbert transform (black) are shown. Persistence duration (gray shadow) is highlighted. (Right) Enlargement of the signals around the stimulus offset.

Detailed illustration of the relation between terminating phases of the stimulus, phase angles of the EEG at the stimulus offset and persistence can be observed in Figure 6 and Supplementary Figure S2. As it can be observed, persistence durations were longer when the stimulus offset coincided with phases towards the troughs of the EEG signal (phases zero and π/2 of the stimulus offset). On the other hand, shorter persistence durations were obtained when the stimulus offset corresponds to the peaks of the EEG signal (phases π and 3π/2 of the stimulus offset).

**Figure 7:**
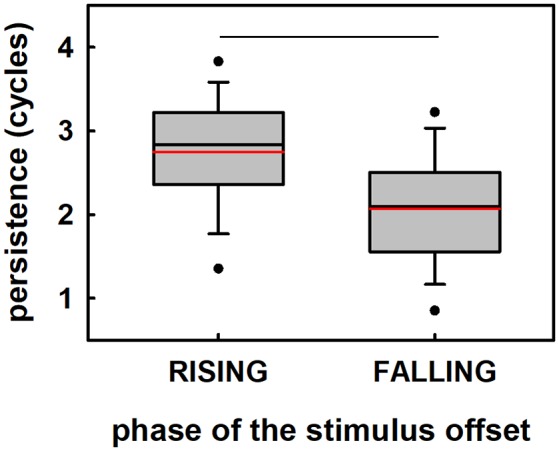
Duration of the persistence of entrainment as a function of the terminating phase of the sinusoidally-varying light (phase of the stimulus at the offset). The effect of rising phases (EEG phases towards the trough of the alpha-oscillation), and falling phases (EEG phases toward the peak of the alpha-oscillation) on persistence is illustrated. Each plot represents the mean (red horizontal line), median (black horizontal line), the 5th and 95 percentiles, and outliers of the persistence durations computed for rising and falling phases (*N* = 19). Statistical significance is highlighted.

A two-way ANOVA was designed to analyze the duration of the persistence of the entrainment (PD) as a function of the terminating phase of the visual stimulation (rising phases and falling phases), and the scalp location (electrode Oz and electrode POz). The two-way ANOVA resulted in a significant effect of the terminating phase of the stimulation on PD so that longer PD was obtained when stimuli ended at ascending phases as compared to that computed when stimuli ended at falling phases (*F* = 37.24, *p* < 0.05; Figure 7). The PD did not vary as a function of the recording electrode (*F* = 3.43, *p* = 0.067). Furthermore, the interaction between factors (terminating phases and recording electrodes) did not have any significant effect on PD (*F* = 0.52, *p* = 0.47).

## Discussion

In this study, the entrainment of EEG oscillations in the alpha frequency band elicited by sinusoidally-varying visual stimulation was described. Specifically, the behavior of the alpha-band entrainment after the end of the visual stimulation was analyzed as a function of the phase of the entrainer at the offset. Our results demonstrated that the phase of the stimulus and the EEG signal at the stimulus offset leads to different persistence durations. When visual stimulation ended at rising phases of the sinusoidal wave (zero, and π/2 the duration of the persistence of the entrainment was statistically significantly longer than that obtained when stimuli ended at falling phases of the sinusoid. Ascending and falling terminating phases of the stimulation corresponded to phases toward the trough and peaks of the EEG oscillation. Based on the dynamical excitation/inhibition framework proposed by Klimesch et al. (2007), our results suggest that different states of the network at the end of the stimulation, corresponding to different states of intrinsic neuronal coupling, may determine the duration of the induced alpha oscillation after the stimulus offset. The source localization analysis suggested that the persistence of the entrainment after the stimulus offset is mediated by the activation of both visual (occipital) and frontal cortical regions.

### Neural Mechanisms of the Persistence of the Entrainment

The results presented in Figures 3C,D are consistent with previous studies demonstrating that periodic visual stimulation induces the entrainment of brain oscillations in the alpha frequency range (Mathewson et al., 2012; De Graaf et al., 2013; Spaak et al., 2014; Notbohm et al., 2016; Kizuk and Mathewson, 2017). The establishment of a robust SSVEP did not occur immediately after the stimulus onset but a few hundreds of milliseconds after (Figures 3C, 6), which is in line with previous findings illustrating that the phase-locking index of the SSVEP increases during the 400 ms following stimulus onset, and stabilize afterward (Wacker et al., 2011). This transition interval can reflect temporal integration processes which result in the recruitment and the progressively increasing synchronization of the neural circuits involved in the generation of the steady-state response (Roß et al., 2002). The fact the SSVEP outlast the visual stimulation (Figures 3C, 6) agrees with previous studies demonstrating that the neural entrainment persists after the stimulus offset for times corresponding with several periods of the entrainer’s stimulus (Sakamoto et al., 1993; Roß et al., 2002; Wacker et al., 2011; Halbleib et al., 2012; Mathewson et al., 2012; De Graaf et al., 2013; Spaak et al., 2014).

Although the persistence of the EEG oscillation after the stimulus-offset has been systematically reported, a possible physiological role of the persistence has not been proposed. The debate has been centered on whether the persistence of entrainment supports any of the most established hypotheses about the origin of SSVEP, which, make disparate assumptions about the nature of the neural oscillations. On the one hand, it has been stated that the SSVEP represents the linear summation of successive transient ERPs, i.e., the superposition hypothesis (Capilla et al., 2011). On the other hand, the SSVEP has been explained as the entrainment of ongoing neural rhythms driven by the periodic presentation of the sensory stimulation, i.e., the entrainment hypothesis (Thut et al., 2011; Mathewson et al., 2012; Notbohm et al., 2016). Alternatively, a third approach does not assume any relationship between the SSVEP and the preferred oscillation frequency of the neuronal population and postulate that a stimulus-driven periodic neural response is added to the ongoing brain activity (Keitel et al., 2014, 2019).

The fact that stimulus-driven brain oscillations in the alpha frequency band are still present when the visual stimulus is no longer delivered (after the stimulus offset; Figures 3C, 6), might be considered as a very strong evidence against the superposition hypothesis. Instead, the aforementioned observation might suggest that the SSVEP results from the entrainment of endogenous alpha rhythm (Spaak et al., 2014).

It is important to note that our experiment was not designed to unravel the origin of the SSVEP. Our choices for using the same stimulation frequency as the IAF were: (i) standardizing the frequency of the stimulation across subjects; and (ii) eliciting SSVEP with the highest possible amplitude by recruiting most of the neural populations tuned to that frequency, i.e., the neural networks responsible for the spontaneous alpha waves, in combination with any neural population sensitive to that frequency that responded to stimulation. Therefore, if the stimulus-driven oscillations reported in this study represent either the entrainment of spontaneous alpha rhythms, frequency-following responses (not necessarily associated with the intrinsic alpha rhythm) or the combination of both, remains in need of further investigations. Nevertheless, our results provide indirect evidence that should be taken into consideration for further discussions.

In this study, an exhaustive description of the transition interval between the stimulus onset and the complete establishment of the SSVEP was not performed. Nevertheless, previous research demonstrated that the decreasing slope of EEG amplitude which characterizes the persistence stage is steeper than the increase in amplitude inherent to the transition period of the steady-state response (Roß et al., 2002; Halbleib et al., 2012). This suggests that different processes underlie the engagement and disengagement of the observed oscillations, which are in turn a consequence of the nonlinear information processing in the human visual system (Halbleib et al., 2012).

Results presented in Figure 4 indicate that both occipital and frontal cortical regions are involved in the generation of the neural entrainment elicited by the alpha visual stimulation. This result is per previous studies suggesting that occipital and frontal cortices interact during the generation of the SSVEP (Halbleib et al., 2012). Those authors, analyzing occipital and frontal scalp regions, observed that once the SSVEP has been established, increased energy in the occipital patch and lower energy in the frontal patch are observed. Other studies also suggest that the engagement of large-scale fronto-occipital cortical networks is a possible mechanism underlying the generation of the SSVEP (Srinivasan et al., 2007; Xu et al., 2013; Li et al., 2015).

Frontal areas obtained in the source localization analysis presented in Figure 4 corresponded to the superior and medial frontal gyri (BA areas 9 and 10, Supplementary Table S2), which have been associated with error processing and detection, as well as with sensory feedback conflict detection (Fink et al., 1999; Gehring and Fencsik, 2001; Mars et al., 2005; Chevrier et al., 2007). Cortical regions responsible for the entrainment persistence resembled those that were active during the presentation of the visual stimuli. This correspondence between the cortical sources of the persistence and the areas involved in the generation of the SSVEP had been previously obtained in EMG studies analyzing the mechanistic role of the alpha oscillatory activity for the temporal organization of visual perception (Spaak et al., 2014). However, unlike the results described in this study, Spaak et al. (2014) only observed activation in occipital areas during the SSVEP or its persistence. Several reasons can account for this discrepancy, including methodological aspects of the studies. Specifically, Spaak et al. (2014) performed source localization analysis of the entrainment by contrasting the current density maps obtained when visual stimuli were presented in the left vs. the right visual hemifields. Therefore, if bilateral frontal activations would have been evident in the row data of Spaak et al. (2014), they would unlikely survive the statistical test.

More importantly, our results demonstrate that, although the cortical regions active during the SSVEP and the persistence of the entrainment were the same, decreased activation in the occipital cortex, accompanied by increased activation of frontal cortical areas were associated with the persistence (Figure 4C). This suggests that the persistence of the entrainment might be an active mechanism triggered by the end of the stimulation rather than the simple reverberation of the SSVEP. The increased frontal activity during the entrainment persistence could be interpreted as an adaptive mechanism processing the lack of expected inputs (elements) in the repetitive sensory stimulation.

This mechanism might contribute to other processes coding temporal expectations, which are not necessarily associated with frontal activations, such as the omitted stimulus-response (OSR). Responses to omitted sensory stimuli within a series, where the omitted stimulus is not necessarily the last in the series, have been extensively reported in retinal ganglion cells (Schwartz et al., 2007; Schwartz and Berry, 2008). OSR is also present in subsequent processing stages of visual information (Karamürsel and Bullock, 1994; Sumbre et al., 2008; Cheng et al., 2014), and has been described using electroretinogram (McAnany and Alexander, 2009) and scalp-recorded visually evoked potentials (Bullock et al., 1994).

Therefore, consistent evidence supports the idea that the persistence of stimulus-driven brain oscillations after the stimulus offset may represent coding mechanisms of temporal expectations. Nevertheless, this perspective must be reconciled with the current view of that entrainment, and its corresponding persistence, represent oscillatory top-down activity modulating perceptual and cognitive processes derived from the presentation of new sensory stimuli.

### The Phase of the Stimulus Offset Modulates the Persistence of Entrainment

Our results demonstrated that rising phases of the stimulus offset facilitated the persistence of entrainment while falling phases impeded it, i.e., they induced longer and shorter persistence duration, respectively (Figure 6). The fact that cortical activation maps in both favorable and unfavorable conditions were similar suggests that the effect of the phase is not mediated by the activation of different cortical networks. Instead, these results could be explained based on the alternation between states of excitation/inhibition measured as oscillations in the EEG recordings (Klimesch et al., 2007; Romei et al., 2007; Mathewson et al., 2011; Klimesch, 2012). In agreement with this hypothesis, it can be suggested that when the EEG oscillation is near its peak values, inhibition is greater and thus, the persistence is shorter. Conversely, when the neural oscillation is approaching the troughs, the persistence is longer.

Rising phases of the stimulus at the offset, which resulted in longer persistence durations, were found to be related to EEG phases near the troughs of the brain oscillation (Figure 5). Conversely, falling phases of the stimulus at the offset were found to be distributed near the positive peaks of the EEG signal and thus turned into unfavorable phases for the persistence of the entrainment (eliciting shorter persistence durations). These results suggest that the phase of the EEG is not only important for the perceptual response to targets presented shortly after the stimulus offset, but for the persistence of the oscillation *per se*. This idea, in conjunction with the possible mechanism involved in the persistence of the entrainment discussed above, suggests that the processing of the lack of expected repetitive sensory stimulus depends on the state of excitation of the neural networks responsible for the entrained oscillation.

Nevertheless, alternative hypotheses need to be considered. They include the possibility that the persistence of the entrainment represents the effect of the intensity (rather than phase) of the stimulus offset on the persistence of the entrainment after the stimulus offset. In other words, stronger stimulus offsets (corresponding with ascending phases) might likely induce longer persistence than weaker stimulus offsets (corresponding with descending phases). For determining the effect of the intensity on PD, a two-way ANOVA was conducted to compare the mean persistence duration as a function of the electrode (Oz, POz), and the terminating phase of the stimulus (zero, π/2, π, and 3π/2), The mean persistence durations obtained at the different terminating phases, and the details of the ANOVA are illustrated in Supplementary Figure S3.

The results of the ANOVA demonstrate that the persistence duration obtained when the stimulus ended at 3π/2 was not statistically different from that obtained when the stimulus ended at π. Likewise, persistence duration obtained when the stimulus ended at phase zero was not statistically different from that obtained when the stimulus ended at π/2. Additionally, persistence durations obtained when stimuli ended at zero can be interpreted as an intermediate between the persistence elicited by π/2, and that obtained when stimuli ended at π and 3π/2. These results strongly support the idea that the phase, rather than the absolute intensity, is the parameter of the stimulus offset which determines the duration of the persistence of alpha-oscillation after the stimulus offset. In other words, the dynamic of the neural activity after the stimulus offset would not only be determined by the stimulus intensity at the offset, but also by the dynamic of the response preceding the end of the stimulation.

Overall, in this study, we provide evidence that the phase information of the stimulus offset and the EEG oscillations are relevant for the functioning of the neural network involved in the persistence of entrainment. Future investigations in this field should take into account the phase of the periodic stimulus offset for the subsequent processing of transient stimuli. Furthermore, our results provided evidence that activation of frontal cortical areas might be involved in the generation of neural entrainment and its persistence. Nevertheless, the role of the distinct cortical areas and the possible mechanisms implicated in the entrainment requires further investigation. The results of these researches may lead to future applications of entrainment as a tool for experimental purposes and the rehabilitation or treatment of neurological disorders.

## Data Availability Statement

The raw data supporting the conclusions of this article will be made available by the authors, without undue reservation, to any qualified researcher.

## Ethics Statement

The studies involving human participants were reviewed and approved by Research and Ethics Committee of the Universidad de Valparaíso (assessment statement code CEC170-18). The patients/participants provided their written informed consent to participate in this study.

## Author Contributions

MO, M-JE, and WE-D contributed to the conception and design of the study. MO and AW contributed to the design and implemented the visual stimulator. MO and PP-G conducted data acquisition and analysis. MO wrote the first draft of the manuscript. All authors contributed to manuscript revision, read and approved the submitted version.

## Footnotes

^1^http://dlnmh9ip6v2uc.cloudfront.net/datasheets/Components/General/YSL-R1042WC-D15.pdf
