# Supplementary material for: Persistence of EEG Alpha Entrainment Depends on Stimulus Phase at Offset
Source: Front Hum Neurosci. 2020 Apr 9;14:139. doi: 10.3389/fnhum.2020.00139 (PMC7161378; doi:10.3389/fnhum.2020.00139)
Supplement: Supplementary file 1 [file Table_1.DOCX]

Supplementary Material

**Table 1.** Cortical regions with statistically significant activations when comparing SSVEP and pre-stimulus.

| BA | LOBE | STRUCTURE |
| --- | --- | --- |
| 18 | Occipital Lobe | Cuneus |
| 18 | Occipital Lobe | Middle Occipital Gyrus |
| 18 | Occipital Lobe | Lingual Gyrus |
| 17 | Occipital Lobe | Inferior Occipital Gyrus |
| 18 | Occipital Lobe | Fusiform Gyrus |
| 19 | Occipital Lobe | Superior Occipital Gyrus |
| 7 | Parietal Lobe | Precuneus |
| 7 | Parietal Lobe | Superior Parietal Lobule |
| 7 | Parietal Lobe | Postcentral Gyrus |
| 19 | Temporal Lobe | Middle Temporal Gyrus |
| 7 | Parietal Lobe | Inferior Parietal Lobule |
| 37 | Temporal Lobe | Inferior Temporal Gyrus |
| 5 | Frontal Lobe | Paracentral Lobule |
| 10 | Frontal Lobe | Medial Frontal Gyrus |
| 10 | Frontal Lobe | Middle Frontal Gyrus |
| 10 | Frontal Lobe | Superior Frontal Gyrus |
