# Supplementary material for: Persistence of EEG Alpha Entrainment Depends on Stimulus Phase at Offset
Source: Front Hum Neurosci. 2020 Apr 9;14:139. doi: 10.3389/fnhum.2020.00139 (PMC7161378; doi:10.3389/fnhum.2020.00139)
Supplement: Supplementary file 3 [file Image_1.PDF]

Supplementary Figure 1

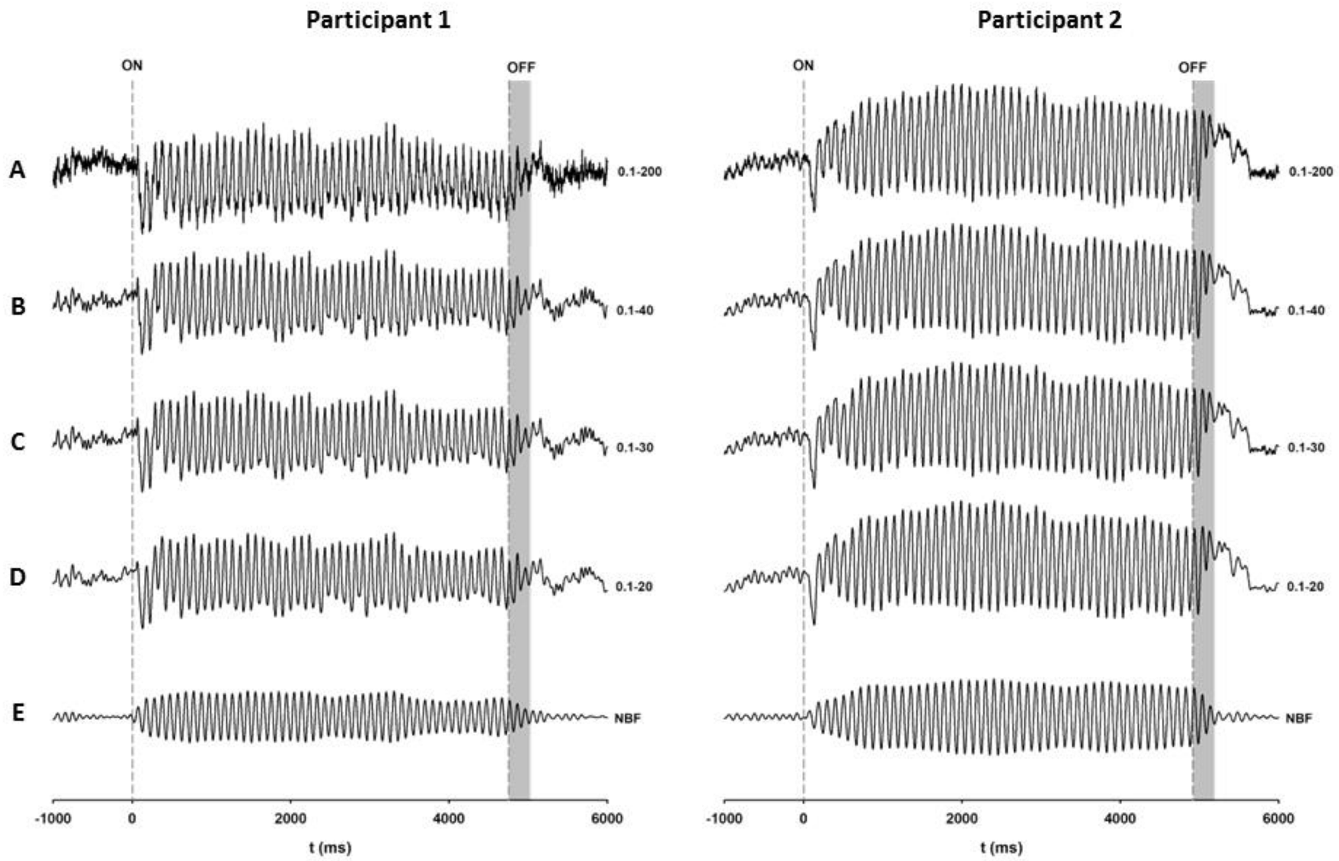

Supplementary Figure 1. Representatives examples of the waveforms obtained in our study. From top to bottom, traces represent the band-pass filtered signal between 0.1 and 200 Hz; the band-pass filtered signal between 0.1 and 40 Hz; the band-pass filtered signal between 0.1 and 30 Hz; the band-pass filtered signal between 0.1 and 20 Hz; and the narrowed band-pass filtered signal around ± 1 Hz of the IAF.
