# Supplementary material for: Persistence of EEG Alpha Entrainment Depends on Stimulus Phase at Offset
Source: Front Hum Neurosci. 2020 Apr 9;14:139. doi: 10.3389/fnhum.2020.00139 (PMC7161378; doi:10.3389/fnhum.2020.00139)
Supplement: Supplementary file 4 [file Image_2.PDF]

Supplementary Figure 2

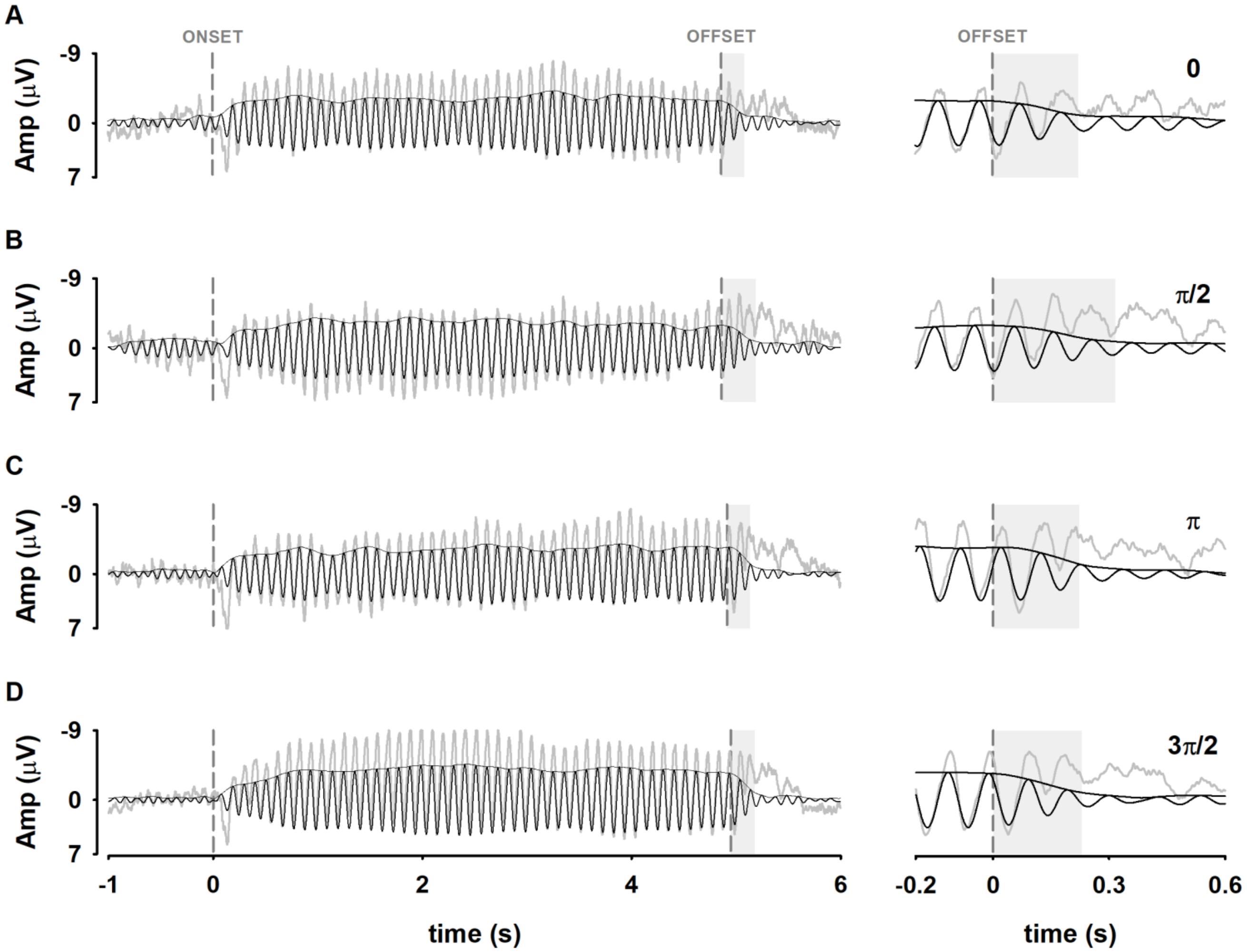

Supplementary Figure 2. Waveforms of the neural entrainment obtained from electrode Oz in one representative subject. (A), (B), (C) and (D) shows waveforms of averaged EEG signals when terminating phase of the stimulus was 0, π/2, π, 3π/2, respectively. (Left) Averaged EEG signals (light gray traces), narrow band filtered signals (black traces) and envelopes calculated from the Hilbert transform (black) are shown. Persistence duration (grey shadow) is highlighted. (Right) Enlargement of the signals around the stimulus offset.
