# Supplementary material for: Persistence of EEG Alpha Entrainment Depends on Stimulus Phase at Offset
Source: Front Hum Neurosci. 2020 Apr 9;14:139. doi: 10.3389/fnhum.2020.00139 (PMC7161378; doi:10.3389/fnhum.2020.00139)
Supplement: Supplementary file 5 [file Image_3.PDF]

Supplementary Figure 3

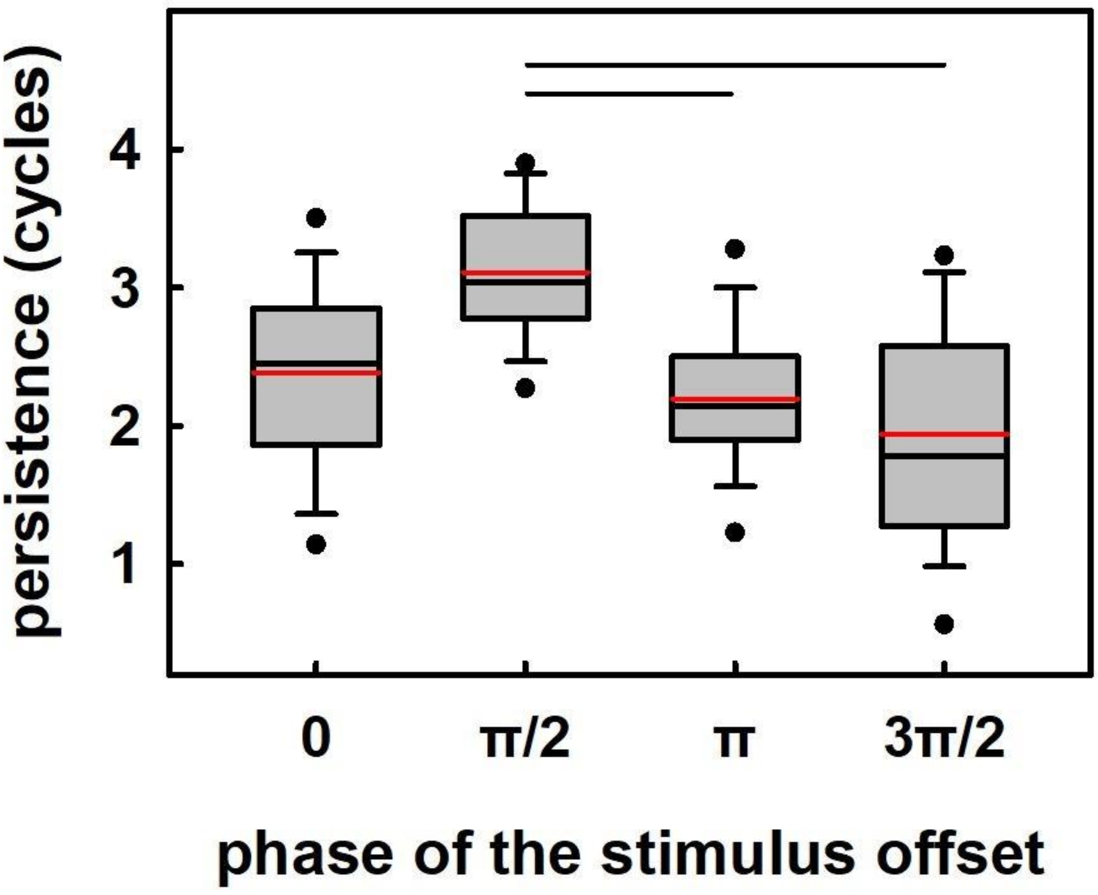

Supplementary Figure 3. Duration of the persistence of entrainment as a function of the terminating phase of the sinusoidally-varying light (phase of the stimulus at the offset). The effect of the ending phase on persistence is illustrated when electrodes Oz and POz were combined. Each plot represents the mean (red horizontal line), median (black horizontal line), the 5th and 95 percentiles, and outliers of the persistence durations computed for each terminating phase (N=19). Horizontal lines highlight terminating phases eliciting statistically significantly different persistence durations (ANOVA: effect of the terminating phase $F=7.49$, $p<0.05$; effect of the electrode $F=1.14$, $p=0.14$, interaction terminating phase x electrode: $F=0.12$, $p=0.95$).
